# Long-term controlled release of ^125^I-tagged BMP-2 by mesoporous bioactive glass with ordered nanopores

**DOI:** 10.3892/etm.2013.1323

**Published:** 2013-09-30

**Authors:** QUAN ZHANG, YE ZHANG, WENJUN CHEN, BINGWEN ZHANG, SHILONG WANG

**Affiliations:** Department of Orthopaedic Surgery, Huashan Hospital Affiliated to Fudan University, Shanghai 200040, P.R. China

**Keywords:** nanometer mesoporous bioactive glass, bone morphogenetic protein-2, delayed release

## Abstract

The aim of this study was to investigate the ability of mesoporous bioactive glass with ordered nanopores (80S MBG) to adsorb and provide the delayed release of ^125^I-tagged bone morphogenetic protein-2 (BMP-2). A 50 mg piece of 80S MBG was produced, which comprised SiO_2_, CaO and P_2_O_5_ in a component molar ratio of 80:15:5. Each MBG piece adsorbed 30 μg ^125^I-BMP-2. Persistent radioactivity in the MBG was periodically measured in simulated body fluid. The total amount of BMP-2 released and the mean amount released per day were calculated. A delayed release curve of BMP-2 was constructed. SPSS 15.0 software was used to perform a statistical analysis. The amount of BMP-2 released in the first two days was one-quarter of the total load. A line equation, y = 490.55×^1/2^ + 7268.82, was obtained from the square root of protein release doses value at 3–94 days. The total amount of BMP-2 released over 94 days was 11.894 μg, which was ~39.6% of the total load. The half-life of the release time was 248 days. From the second week, the rate of BMP release had stabilized to a mean of 37.42±18.67 ng/day and the difference of the mean amount released per day had no statistical significance (P>0.05). High adsorption and delayed release effects of BMP-2 were observed in 80S MBG. The delayed release conforms to the Higuchi equation, which indicates possible applications in promoting bone healing.

## Introduction

Methods for the repair of bone loss include the filling of bone tissues and the replacement of bone materials. The participation of protein factors promotes bone formation. In recent years, the clinical application of artificial bone materials, including calcium hydroxyapatite, tricalcium phosphate and bioactive glass, has been studied (1–4). In addition to mediating ossification, ideal bone replacement materials may also store and release (delayed release) active substances required for bone formation. Therefore, an effective concentration of activity proteins in the filled area may promote the repair of bone disunion and loss. Several scholars have used synthetic materials, including calcium hydroxyapatite, tricalcium phosphate, calcium sulfate and bioactive glass with activity protein factors, including bone morphogenetic proteins (BMPs) and transforming growth factor, to repair bone loss (5–7). In addition, a number of scholars have integrated drugs, such as Ag^+^ and antibiotics, into bone filler materials to cure osteomyelitis and to study the controlled release process (8,9). Therefore, active drugs may be provided in infected areas for a longer time period and the effectiveness of osteomyelitis treatment may be enhanced (10–12). However, few researchers have studied the long-term delayed release of bioactive glass-adsorbed BMPs (13).

BMPs are special bone growth factors that may induce the formation of bone and cartilage *in vivo* and *in vitro*. BMP-2 and BMP-7 have high ossification activities and are used extensively in bone repair (14–16). In solution, BMP-2 flows and diffuses easily *in vivo*. However, BMP-2 is also easily degraded by proteases and is unable to produce a long-term marked effect in local areas, thereby affecting the effectiveness of the treatment. The use of BMPs in bone repair involves loading active BMPs with sufficiently high purity into a bone fill material with delayed release properties, and implanting the material *in vivo*. The BMPs are released by degradation and replaced with bone material. BMPs enhance bone formation when maintained at a high concentration in local areas for a long time period.

**Figure 1:**
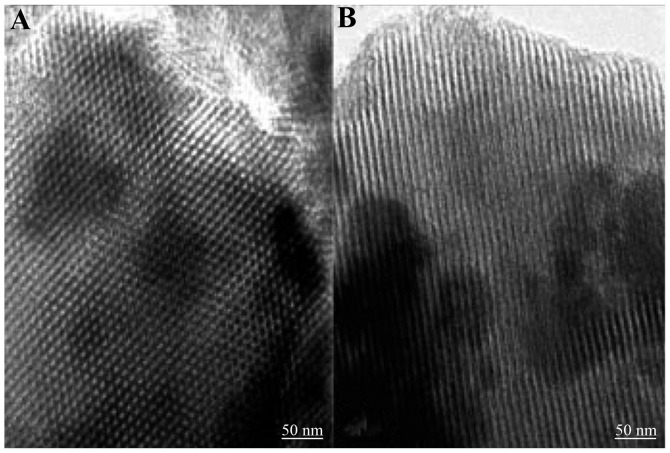
Microstructure of 80S MBG (TEM). (A) Horizontal cross section; (B) vertical cross section. Regular mesopores with a diameter of ~5 nm were observed in 80S MBG. TEM, transmission electron microscopy; MBG, mesoporous bioactive glass.

Bioactive glass has good biocompatibility and surface bioactivity. Such material has dual mechanisms of action, specifically, the induction and mediation of ossification. Bioactive glass is a suitable material for the repair and replacement of lost bone. A mesoporous bioactive glass with ordered nanopores (80S MBG) was manufactured by Yan in 2005 (17). 80S MBG has pores with a diameter of 5–20 nm, a specific pore volume of 0.40 cm^3^/g and specific surface area of 300 m^2^/g (Fig. 1). This material has the ability to absorb and delay the release of active proteins, and also has good bioactivity and tissue compatibility. 80S MBG promotes ossification by modulating IGF-11 gene expression in ossification cells (18,19). The current study aims to: i) test the efficacy of delayed release and adsorption of BMP-2 by 80S MBG, ii) investigate the effective association of MBG and BMPs, and iii) collect information concerning the delayed release of BMP-2 by 80S MBG relevant to its use as a filler for bone loss, for stimulating and inducing ossification, effectively aiding fracture healing disorders and repairing bone loss.

## Materials and methods

### Characteristics of 80S MBG

The component molar ratio of 80S MBG (provided by the Inorganic Chemistry Teaching and Study Department of Fudan University, Shanghai, China) was 80:15:5 (SiO_2_:CaO:P_2_O_5_). The ordered mesopores were 5–20 nm. The 80S MBG appeared gray in color. MBG (50 mg) was made into a tablet with diameter of 4 mm, depth of 3.5 mm. This material was composed of microparticles with a diameter of 38–76 μm.

### Preparation and identification of ^125^I-rhBMP-2

#### Tagging

To one Iodogen test tube (Sigma, St. Louis, MO, USA) was added 20 μg/10 μl recombinant human BMP (rhBMP)solution (Dahui Bioengineering Company, Guangzhou, China; purity >95%) and 1.5 μl Na ^125^I solution (365 μCi; Radiomedicine Institute, Shanghai, China). After 10 min of mixing and reaction at room temperature, the reaction was terminated by the addition of a 0.01 mol/l pH 7.4 phosphate-buffered saline (PBS) solution. Samples were obtained when the tagging reaction had been performed for ~10 min. A silica gel instant thin-layer chromatography paper (ITLC/SG) method was used to analyze the tagging rate. The radioactive tagging rate obtained was >90%.

#### Purification

The aforementioned reaction liquid was purified via Sephadex G25 column chromatography. One chromato bar (Sigma Chemical Co., St. Louis, MO, USA) filled with Sephadex G25 gel was used, with 0.01 mol/l PBS and pH 7.4 buffer solution for elution and purification. After repeating the elution and purification twice, a high purity ^125^I-rhBMP-2 protein tagging solution was obtained. This solution was analyzed, identified and quantified (radiochemical purity >95%) by ITLC/SG. Following aseptic filtration of 0.22 μm, a concentrated solution of ^125^I-rhBMP-2 was obtained.

#### Identification

i) ITLC/SG method: ITLC/SG was used as the vehicle. After spreading with alcohol and water (ratio, 85:15 v/v), each chromatography paper was cut into small strips with an equal width of 1 cm. The strips were placed in the radioactive measurement test tube for assay. The Rf value of ^125^I-rhBMP-2 protein was 0.0. The Rf value of ^125^I was 0.7–0.8. The radioactivity percentage binding rate (Rf value = 0.0) was the radiochemical purity of ^125^I-rhBMP-2 protein. ii) Trichloroacetic acid (TCA) precipitation method: a 100-μl solution of ^125^I-rhBMP-2 protein was placed in a small centrifuge tube and 1.5 ml 10% aqueous TCA solution was added. After mixing, the solution was centrifuged for 5 min at 686 × g. The supernatant was removed and the radioactivity percentage binding rate (TCA sediment) was tested. The result indicated the radiochemical purity of the ^125^I-rhBMP-2 protein.

Following purification with Sephadex G25 by column chromatography, the radiochemical purity of the ^125^I-rhBMP-2 protein was evaluated and a result of ≥95% was obtained.

### Radioactive measurement

The gauge used was an SH-682 radioimmunity γ-ray counter (Shanghai Nucleus Institute Rihuan Apparatus No. 1 Factory, Shanghai, China). A sample applicator was placed at the bottom of a 12×60 mm plastic tube for radioimmunity measurement. Radioactive counts per min per sample were measured using the SH-682 radioimmunity γ-ray counter.

### Loading of quantitation protein on 80S MBG

A tablet of MBG was placed at the bottom of each 12×60 mm plastic tube for radioimmunity measurement (6 MBG material tubes). A ~15 μl aliquot of ^125^I-rhBMP-2 protein solution (rhBMP-2 protein concentration, 2 mg/ml; load protein quantity, 30 μg/15 μl/tablet) and was added to the center of each performing. After dehydration for three days on a 4°C drying dish, the samples were stored for further use.

### Delayed release test

The groups were as follows: test group, MBG-^125^I-rhBMP-2, n=6 and control group, MBG blank, n=4. The dehydrated 80S MBG material preforming (preforming/tube) loaded with ^125^I-rhBMP-2 protein was obtained. Simulated body fluid (SBF) buffer solution (1 ml) was added to each tube. The tubes were then placed in a 37°C water bath. After a specific interval (times are provided in Tables I and II), the liquid was obtained. A 2 ml SBF buffer solution was used to wash the preforming solid (twice). Persistent radioactivity [radioactivity count/min (cpm)] in the preforming solid was tested. The mean dose released per day was obtained after correction for attenuation using the following formula: (Measured value at present time-point - measured value at previous time-point)/release days.

### Construction and analysis of a delayed release curve

The total amount of rhBMP-2 released and the mean dose released per day at each time-point in the test and control groups were used to plot a rhBMP-2 delayed release curve. The character was analyzed and a general equation for the delayed release of BMP-2 was determined according to the curve.

### Statistical analysis

SPSS 15.0 software (SPSS, Inc., Chicago, IL, USA) was used to analyze the data, which were expressed as mean ± standard deviation. One-way ANOVA was used o perform group comparisons. Partial data were used for further comparison among means [least significant difference (LSD)]. P<0.05 was considered to indicate a statistically significant result.

## Results

### Amount of ^125^I-rhBMP-2 released by 80S MBG per day

**Figure 2:**
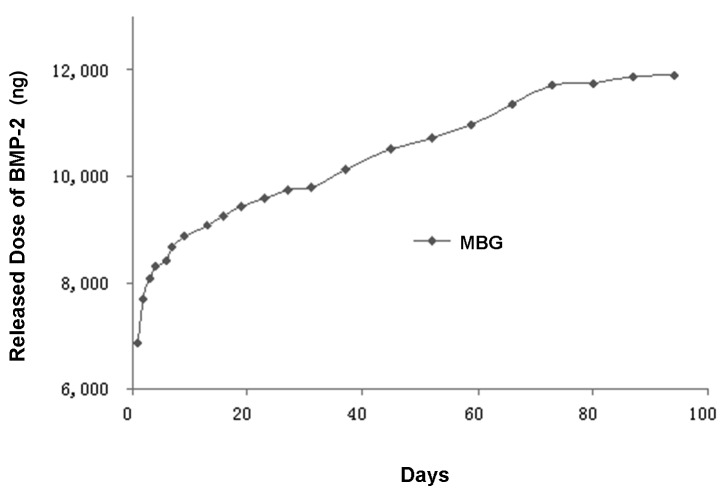
Cumulative released dose of BMP-2. In the first three days, the released dose of BMP-2 was relatively large and the gradient of the curve was steep. The rate of BMP-2 release decreased from 13 days onwards and the gradient of the curve became gradually less steep. The total released dose increased steadily after 10 days. BMP-2, bone morphogenetic protein-2; MBG, mesoporous bioactive glass.

Table I and Fig. 2 show the cumulative amount of ^125^I-rhBMP-2 released at each time-point. The mean amount of ^125^I-rhBMP-2 released each day is listed in Table II. The amount released during the first day was 6.9 μg, which was 23% of the total load. The amount released during the second day was 0.8 μg, which was 2.7% of the total load amount. From the third day, a period of relatively steady delayed release was achieved. By the 94th day, the total amount released was 11.9 μg, which was 39.7% of the total load.

**Figure 3:**
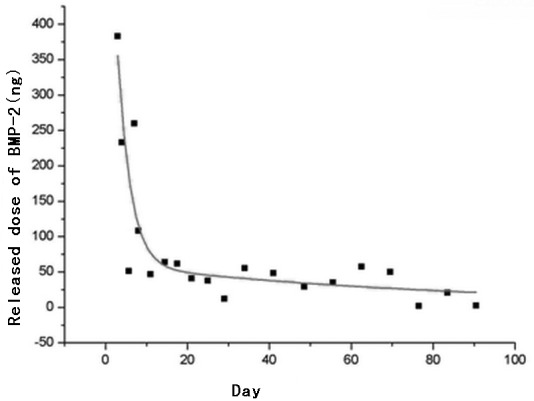
Mean dose of BMP-2 released by MBG per day. After 10 days of release, the rate of release was maintained at ~12 ng/day and the released dose was fairly steady. BMP-2, bone morphogenetic protein-2; MBG, mesoporous bioactive glass.

According to the time curve of the amount of ^125^I-rhBMP-2 released per day (Fig. 3), the amount released per day was stable at ~2–64 ng/day after 10 days of release. The mean amount released was 37.42±18.67 ng/day and the curve was steady. ANOVA-LSD analysis (Tables III and IV) show that the mean amount released per day had no significant differences after 10 days (P>0.05), which indicated that the amount of ^125^I-rhBMP-2 released per day was steady.

### 50% delay in release time of rhBMP-2 by 80S MBG

**Figure 4:**
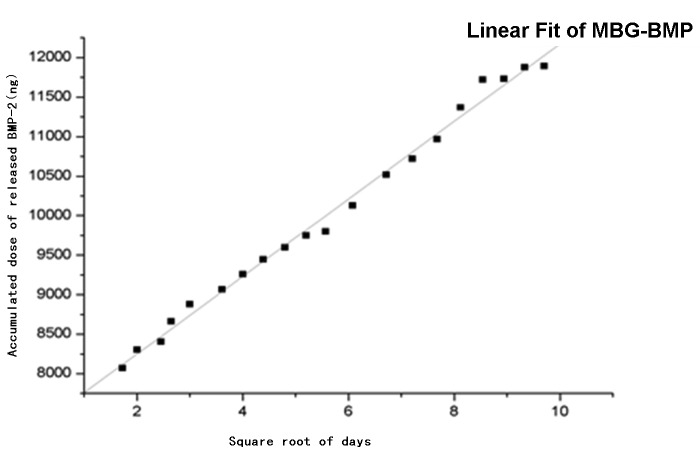
Square root of MBG total released dose at days 3–94. Square root (x^1/2^) of release dose (y) and release day times accorded with the following equation of straight line: y = 490.55×^1/2^ + 7268.82. MBG, mesoporous bioactive glass; BMP-2, bone morphogenetic protein-2.

The square root of the amount of protein released by 80S MBG at days 3–94 was used to create a scatter diagram. A line was constructed using linear regression analysis (Fig. 4). The equation of the line is as follows: y = 490.55×^1/2^ + 7268.82 or × = [(y - 7268.82)/490.55]^2^. The calculation shows the half-life of 30 μg BMP-2 loading, where × = [(15,000 - 7,268.82)/490.55]^2^ = 248.38 days.

## Discussion

80S MBG has ordered nanometer-sized mesopores and a large pore volume and specific surface area. This material is expected to have a good adsorption function. Aside from the air space between particles, the large pore volume and specific surface area will attach more BMPs and further increase the enduring load ability for BMPs of MBG. In SBF, recrystallization may be observed on the surface of MBG through rapid ion exchange. Hydroxyapatite (HA) crystal coatings are produced on the surface of MBG materials or at the cavosurface of air spaces (18,20–22), which makes the aperture of the lumens in the micropore structure zoom out and the proteins inside cannot be separated quickly. These types of ‘self-close’ features may contain activity proteins *in vivo* and delay their release with the degradation of the material. The test of long-term controlled release confirmed that 80S MBG possesses the ‘self-close’ attribute, along with good adsorption capacity and delayed release ability for BMPs.

Initially, BMP-2 was generated by a quick-release process, in which 6.9 μg was released during the first day and 0.8 μg was released during the second day. A total of 25.67% of the adsorbed protein was released within two days. This observation may be attributed to the adsorption of BMP-2 by 80S MBG. Furthermore, surface adhesion of the protein to the material was weak and thus the protein was easily separated in the SBF. An amount of HA crystals sufficient to delay the release of BMP-2, i.e., the ‘self-close’ features that inhibit the absorption of the protein *in vivo*, had not yet formed on the surface of the MBG. Dissolution and precipitation of surface adsorption proteins are the most significant processes of the rapid release during the initial stage.

From the third day, the release rate markedly slowed down. From day 10 to 3 months after the initial observation, the release of BMP-2 was relatively steady and the mean dose released per day was maintained between 2 and 64 ng. The mean amount released per day had no significant differences after 10 days (P>0.05). This type of release is associated with the particular attributes of MBG. After the third day, crystal growth reconstruction on and within the surface of the MBG tablet material was completed, leading to a typical porous delayed release behavior of the vehicle. The Higuchi equation (23) expresses the delayed release of a drug by a porous vehicle as: M_t_ = AM_0_ [C_s_ D_eff_ (2C_d_ - ɛC_s_) t]^1/2^, where M_t_ is the dose of drug released in time t; M_0_ is the total amount of loaded drug; A is the surface area of the vehicle material; D_eff_ is the effective solubility factor of drug in the vehicle micropores; C_s_ is the drug solubility; C_d_ is the drug concentration and ɛ is the microporosity of the vehicle. The release behavior of a drug in a porous delayed release vehicle should be in accordance with the equation. For a known vehicle material and loaded drug (e.g., 80S MBG and ^125^I-BMP-2 in this study), M_0_, C_s_, D_eff_, C_d_ and C_s_ are all constant, whereas A and ɛ are also fixed at a specific value following the reconstruction of the MBG material surface. Therefore, this finding is the most direct and simple manifestation of the equation indicating that M_t_ and t^1/2^ are directly proportional. In the current study, the square root of time (days) and BMP-2 released dose exhibited a linear correlation, that is, the Higuchi model almost exhibits a straight line. This indicates that the release behavior of ^125^I-BMP-2 by 80S MBG is in accordance with the Higuchi equation (24–26).

The adsorption and delayed release behavior of BMP by MBG has a significant contribution to the repair of fracture healing disorders and bone loss. MBG is known for demonstrating efficacy in inducing and mediating ossification. After being used as a filler to replace the lost bone, the MBG is degraded *in vivo* and is gradually replaced by bone tissues. The presence of BMP-2 further enhances ossification. However, the repair of bone loss and the curing of non-union is a long-term process. BMP-2 may produce a marked effect and it must be maintained at a high concentration for a long time period. Uludag *et al*(27) observed that the BMP concentration in a local area is closely associated with the ossification-inducing potential, thus demonstrating their direct correlation. In summary, a higher concentration of BMP corresponds to a stronger ability to induce ossification of the surrounding osteoblasts (27). The local delayed release of BMP induces ossification and prevents neoplasm-like changes caused by exorbitant or ectopic ossification resulting from BMP moving out of the treated area. The study by Valentin-Opran *et al*(28) shows that a physiological concentration of BMP-2 (2 ng/ml) in a raw state may be sufficient to complete bone repair. The analysis of the mean released dose per day in Table II shows a relatively rapid release in the first 10 days. This corresponds with the hematoma formation period during fracture repair and reconstruction. The release of a high concentration of BMP-2 by the vehicle is favorable to chemotaxis and the accumulation of stem cells in the blood and in the parenchyma. After two weeks, the protein release rate of the material may be observed to be steady at ~40 ng and this rate is maintained for two months. During this stage, bone union enters into the hematoma organization stage; a metastable BMP-2 concentration is favorable for the further proliferation and cell differentiation of ossification cells, which are similar to osteoblasts. After 73 days, the released amount further decreases. Within three weeks after the continuous decrease, the approximate mean of the amount released/day was 8 ng, which corresponds with the advanced stage of bone repair. The demand of the organism for cytokines is reduced; thus, a decreased released dose may prevent ectopic ossification and neoplasm-like changes, whilst continuously inducing ossification and promoting repair. After 248 days, or 8 months, the amount of BMP-2 retained by 80S MBG, as derived by the Hugichi equation, had dropped by 50%, further indicating that the delayed release function of MBG facilitates the long-term maintenance of an effective BMP-2 concentration in the filling area, thereby meeting clinical practice requirements (29).

In summary, 80S MBG exhibits good adsorption and delayed release effects for BMP-2. The delayed release characteristics conform to the Higuchi equation. During the most important first three months of bone healing, an effective concentration of BMP-2 control released may enhance bone ossification. This result indicates that 80S MBG is of significant value in various clinical areas, including tissue engineering, the controlled release of drugs, dentistry, orthopedics and oral and maxillofacial surgery. However, in this study, SBF was used as the release system of the test of 80S MBG. Although its ion concentration and structure are completely in accordance with those of body fluids, SBF is far from the true body fluid. The release action *in vivo* occurs in a more complex and multivariate process. The delayed release of one type of protein from the vehicle may also have an effect on other proteins and cell tissues in body fluid. Therefore, further tests *in vivo* are required.

## Figures and Tables

**Table I:** Cumulative dose of BMP-2 protein released by MBG at different time-points (n=6).

Time (days)	Mean cumulative released dose (ng)	SD	Percentage of the total load
1	6,864	314	22.88
2	7,688	334	25.63
3	8,071	376	26.90
4	8,304	387	27.68
6	8,404	394	28.01
7	8,664	365	28.88
9	8,880	388	29.60
13	9,068	399	30.23
16	9,259	446	30.86
19	9,444	463	31.48
23	9,600	423	32.00
27	9,750	415	32.50
31	9,799	434	32.66
37	10,130	398	33.77
45	10,516	385	35.05
52	10,721	380	35.74
59	10,968	379	36.56
66	11,370	302	37.90
73	11,721	306	39.07
80	11,734	215	39.11
87	11,877	297	39.59
94	11,894	246	39.65

BMP-2, bone morphogenetic protein-2; MBG, mesoporous bioactive glass.

**Table II:** Mean dose of BMP-2 protein released per day by MBG at different time-points (n=6).

Time (days)	Mean dose released per day (ng)	SD
1	6,864	314
2	824	80
3	384	55
4	232	29
6	50	5
7	260	177
9	108	51
13	47	10
16	64	36
19	61	35
23	39	17
27	38	10
31	12	15
37	55	7
45	48	6
52	29	8
59	35	6
66	57	15
73	50	6
80	2	31
87	20	34
94	2	25

BMP-2, bone morphogenetic protein-2; MBG, mesoporous bioactive glass.

**Table III:** ANOVA of the groups.

Comparison	Sum of squares	df	Mean square	F	Significance
Between groups	264917816.403	21	12615134.114	1856.171	<0.001
Within groups	747595.160	110	6796.320		
Total	265665411.563	131			

**Table IV:** Multiple comparisons for dependent variables: released dose/day (least significant difference).

(I) Day	(J) Day	Mean difference (I–J) (lower bound)	Standard error (upper bound)	Significance (lower bound)	95% Confidence interval	

					Lower bound	Upper bound
13	1	−6817.255^a^	47.597	<0.001	−6911.580	−6722.930
	2	−776.655^a^	47.597	<0.001	−870.980	−682.330
	3	−336.810^a^	47.597	<0.001	−431.135	−242.485
	4	−185.230^a^	47.597	<0.001	−279.555	−90.905
	6	−5.072	47.597	0.907	−98.537	87.649
	7	−213.460^a^	47.597	<0.001	−307.785	−119.135
	9	−61.078	47.597	0.202	−155.404	33.247
	16	−16.943	47.597	0.723	−111.269	77.382
	19	−14.482	47.597	0.762	−108.807	79.844
	23	7.888	47.597	0.869	−86.437	102.214
	27	9.213	47.597	0.847	−85.112	103.539
	31	34.808	47.597	0.466	−59.517	129.134
	37	−8.323	47.597	0.862	−102.649	86.002
	45	−1.258	47.597	0.979	−95.584	93.067
	52	17.662	47.597	0.711	−76.664	111.987
	59	11.558	47.597	0.809	−82.767	105.884
	66	−10.477	47.597	0.826	−104.802	83.849
	73	−3.240	47.597	0.946	−97.565	91.085
	80	45.007	47.597	0.346	−49.319	139.332
	87	26.517	47.597	0.579	−67.809	120.842
	94	44.490	47.597	0.352	−49.835	138.815

a A significant difference (P<0.05).
